# Primary Spontaneous Inferior Clival Cerebrospinal Fluid Leak

**DOI:** 10.7759/cureus.17967

**Published:** 2021-09-14

**Authors:** Christian T Ogasawara, Kurtis Young, Princess Jhoy Bonilla, Thomas Noh, John Cho

**Affiliations:** 1 Surgery, John A. Burns School of Medicine, University of Hawaii at Manoa, Honolulu, USA; 2 Surgery, University of Hawaii, Honolulu, USA; 3 Neurological Surgery, Henry Ford Hospital, Detroit, USA; 4 Otolaryngology - Head and Neck Surgery, Straub Medical Center, Honolulu, USA

**Keywords:** clivus, inferior, spontaneous csf leak, endoscopic endonasal approach, meningitis

## Abstract

Primary, spontaneous cerebrospinal fluid (CSF) leaks secondary to defects in the clivus are exceedingly rare. Additionally, primary, spontaneous CSF leaks are typically present in obese women with idiopathic intracranial hypertension (IIH). In the present study, we report the first case of a primary, spontaneous CSF leak in the inferior-posterior wall of the clivus in an atypical patient with a BMI of 18.9 kg/m^2^ without IIH. Accurate diagnoses of CSF leaks are imperative in the context of preventing meningitis, and delays in diagnosis and treatment are associated with worse outcomes. Improved characterization of rare, spontaneous CSF leaks may prove beneficial in correctly diagnosing affected patients.

## Introduction

Cerebrospinal fluid (CSF) leaks have been well established as a risk factor for bacterial meningitis [1]. Accumulated CSF in the paranasal sinuses may serve as a nidus for retrograde microorganism transport through defects in the skull base. Although the most common causes of CSF leak include trauma (80%) and iatrogenic injury (16%), around 5 per 10,000 people experience spontaneous CSF leaks each year [2]. Due to the rare occurrence of spontaneous CSF leaks, accurate diagnoses and treatment are often delayed [3]. This is of particular significance when considering that the mortality of bacterial meningitis may be as high as 15% in the United States [4]. Although the underlying etiology behind spontaneous CSF leaks is poorly understood, the majority of patients are middle-aged or older females with elevated BMIs [5-7]. Additionally, many of these patients present with elevated intracranial pressures, with opening CSF pressures being between 25 and 27 cmH_2_O [8,9]. This phenomenon has led to some positing a possible relationship between idiopathic intracranial hypertension (IIH) and primary spontaneous CSF leak [7,10].

There are conflicting reports regarding the location of the bony defect causing spontaneous CSF leak. In a case series of six patients with clival CSF leaks, all defects were found to be in the superior, midline area of the clivus [6]. Several small retrospective reviews determined the cribriform plate or ethmoid roof as the most common locations for defect [5,7]. In a retrospective case series of 56 individuals with spontaneous CSF leaks, the most common bony defects were found in the lateral sphenoid sinus (41%), ethmoid roof (30%), cribriform plate (21%), central sphenoid (12.5%), and frontal sinuses (12.5%) [9]. In the largest study to date of 105 patients, the most likely sites for CSF leak included the cribriform plate (51%), sphenoid lateral pterygoid recess (31%), and ethmoid roof (8%) [8]. In the present study, we present a rare case of primary spontaneous CSF leak in a 54-year-old female with a negative prior β2-transferrin assay. To the best of our knowledge, this is the first case reported in the literature of a spontaneous CSF leak secondary to a defect in the posterior-inferior clival wall in a patient with an atypical BMI of <25 kg/m^2^.

## Case presentation

A 54-year-old female with no prior history of surgery or trauma presented to the emergency department with a chief complaint of severe headache for the past two days. She never experienced an episode like this in the past, but did report intermittent clear, thin liquid leaking through the right side of her nose for the past 15 months. She reported that the rhinorrhea was worse in the morning and that she could feel the fluid spread down her throat while upright. Additionally, tipping her head forward would elicit a significant amount of clear fluid discharge from her nose. The prior year, the discharge became thicker, and she subsequently visited an otolaryngologist. The right-sided nasal discharge was collected and analyzed, and the β2-transferrin assay was negative for CSF. She was given a diagnosis of chronic sphenoid sinusitis versus CSF leak at this time.

**Figure 1:**
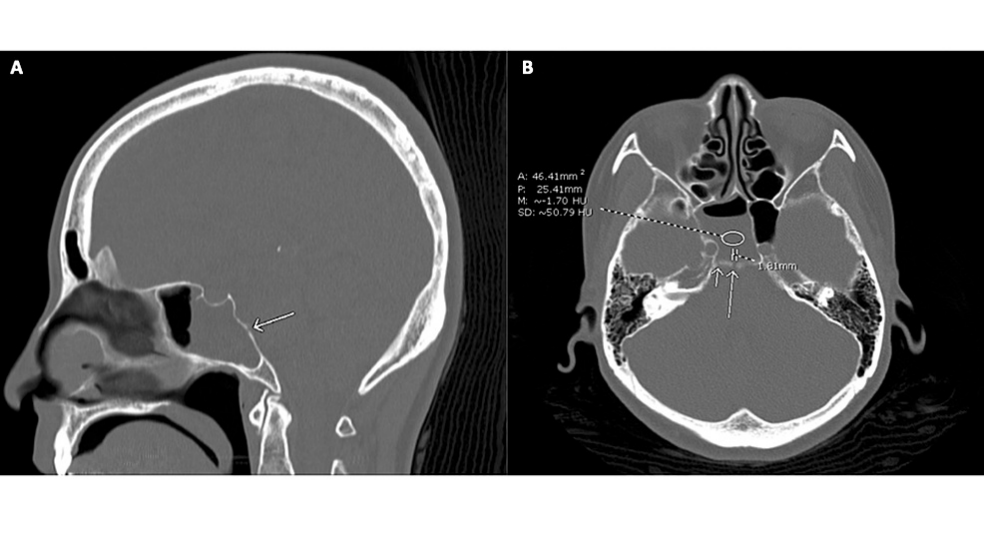
A. Sagittal CT head demonstrating the right clival defect (arrow) and small air-fluid level in right dominant sphenoid sinus. B. Axial CT head demonstrating small air-fluid interface in the right dominant sphenoid sinus, and 1.8 mm (long arrow) and 1 mm (short arrow) defects along the posterior wall of the right sphenoid sinus CT, computerized tomography.

The initial computerized tomography (CT) scan ordered in the emergency department revealed a small air-fluid interface in the right dominant sphenoid sinus, suggestive of sphenoid sinusitis (Figure 1).

The patient was discharged home on a regimen of Augmentin, Percocet, Sudafed, and Zofran. However, the patient’s condition failed to improve the following day, and she continued to exhibit symptoms of waxing and waning confusion, delirium, and hallucinations. She was brought back to the emergency department, for further workup and management. Laboratory findings were significant for a neutrophil-predominant leukocytosis (14.4 x 10^9^/L) and hyponatremia (127 mmol/L). The patient’s lumbar puncture yielded a colorless, hazy fluid with elevated protein (86 mg/dL) and total nucleated cells (1,194/µL with 77% segs). These findings in conjunction with the patient’s co-existing acute sinusitis were suggestive of bacterial meningitis, and she was subsequently admitted to the inpatient floor, receiving antimicrobial coverage with IV vancomycin, ceftriaxone, and acyclovir.

The following day, the patient was assessed via high-resolution CT sinus, confirming the presence of moderate to large amounts of fluid in the right dominant sinus. Alarmingly, the fluid accumulation seemed to be more pronounced than that on admission, and some mild mucosal thickening was noticed on the ethmoid and maxillary sinuses. It is important to note that a 2-mm lucency was found involving the posterior wall of the sphenoid sinus, indicative of a bony defect or erosion. The patient was then informed and counseled regarding the possibility of surgery versus alternative management, and she consented to undergo image-guided endoscopic repair of her CSF leak. During the operation, the small, dehiscent area with granulation tissue along the posterior-inferior clival wall was found. The defect was repaired with a generous nasal septal flap, using a branch of the sphenopalatine artery as the pedicle. After applying fibrin glue to the septal flap, a lumbar drain was placed. The procedure was successful, with no untoward events noted during the operation.

The rest of the patient’s hospital course was uneventful, and repeat CSF analysis demonstrated a marked improvement in total nucleated cells from 1,194/µL at admission to 102/µL. Additionally, her CSF protein counts had returned back to normal limits. Additionally, the patient’s WBC count continued to downtrend and was within normal limits on discharge, which were well correlated with significant improvement in her initial symptomology. The patient was discharged with no additional concerns on follow-up visits at one week, one month, and three months.

## Discussion

The clivus is a bony structure at the central, posterior skull base that forms when the body of the sphenoid bone, the basisphenoid, combines with the basal portion of the occipital bone, the basioccipital, at the sphenooccipital synchondrosis. Its posterior surface is situated anteriorly to the pons and medulla oblongata [11]. At the level of 28 mm and 11 mm rostral of basion the thickness of clivus was 18.3 mm and 9.3 mm, respectively [12]. This may explain why the defect in the present case was found in the inferior segment of the clivus, which is associated with conventionally thinner bone. However, this same rationale highlights how unusual it is that many CSF leaks in the clival wall are more superiorly located [6].

The etiology behind our patient’s posterior-inferior clival CSF leak is unclear, but there are several theories that may provide additional insight. Although the exact pathophysiology of spontaneous CSF leaks in any location have not been fully elucidated, there are three factors that are essential: a bony defect, a tear in the dura, and a pressure gradient. Fossa navicularis magna (FNM), canalis basilaris medianus (CBM), and craniopharyngeal canal are rare anatomical variants of the clivus [13]. These variants are noted to have communicating tracts from the nasopharynx to the skull base [13]. It has been noted that FNM and CBM serve as tracts for intracranial infections and although, to our knowledge, there have been no associated cases of spontaneous CSF leak, these variants could possibly be a contributing factor [13]. Faizuddin Ahmad et al. postulated that excessive pneumatization of the sphenoid sinus causes a thin bony wall at some points of clivus and sphenoid [14,15]. Continuous pressure pulses of CSF and arterial pulsations can wear down the clivus, particularly in cases where the sphenoid sinus is markedly pneumatized. Furthermore, all recorded cases of clival CSF leaks have occurred in adults, and this is of particular significance since maximum CSF pressure and pressure waves are attained in adulthood. Additionally, since the patient characteristics associated with spontaneous CSF leaks and IIH are so similar, several authors have hypothesized that spontaneous CSF leaks may be a variant of IIH [7,10,16,17]. The importance of increased intracranial pressure in spontaneous CSF rhinorrhea is also evidenced radiologically by frequently observed empty sella syndrome (80%) and arachnoid pits (63%) and a thinned and broadly attenuated skull base [18]. Clival CSF leaks have been seen in congenital bone malformations such as Marfan’s syndrome [19]. Marfan’s syndrome has been implicated in the pathogenesis of clival CSF leaks through associated weakness of the dura and meningeal diverticulae, which could predispose to spontaneous CSF leaks [11]. However, the patient presented in this case had a BMI of 18.9 kg/m^2^, which is also atypical and expressed no prior symptoms suggestive of IIH. She also has no past medical history that would predispose her to congenital bone malformations or weaknesses in the dura. Instead, the most probable explanation for the patient’s spontaneous CSF leak may be attributable to the excessive pneumatization of the right dominant sphenoid sinus and the subsequent thinning of the inferior clival bone, which may have been exacerbated by her prior history of osteoporosis. 

Early detection is essential in preventing the severe sequelae of CSF fistulas, including bacterial meningitis and intracranial abscess [20]. The patient presented with symptoms consistent with a CSF leak. Four months later, she was tested for CSF leak via a β2-transferrin assay, which has a sensitivity and specificity of 94-100% and 98-100%, respectively [21,22]. However, several factors may influence the efficacy of the β2-transferrin assay including inflammatory conditions such as bacterial meningitis [23,24]. During the time that her CSF was collected, the otolaryngologist had suspected her to have chronic sinusitis. It is quite possible that the patient could have had concomitant subclinical meningitis at this time. Regardless, the final diagnosis given to the patient was chronic sinusitis versus CSF leak. Due to the potential severe complications of CSF leak, all patients with this suspected condition should be considered for further workup. High-resolution CT scan is the first-line imaging modality when searching for skull base defects [25]. When unclear, contrast agents may be applied in the thecal sac via lumbar puncture. The patient is then tilted head-down, and asked to perform maneuvers to provoke CSF leakage (head hanging or Valsalva). Another CT scan is taken at this time, in a process is known as CT cisternography [26]. It is likely that the patient’s posterior-inferior clival defect could have been detected earlier if this were done, effectively preventing her from developing bacterial meningitis in 2019. It is important to note that while bacterial meningitis secondary to CSF leak may develop in as many as 15% of cases, this number dramatically increases to as high as 47% in cases where a clival defect is involved [18]. Hence, it may be prudent for clinicians to more aggressively workup patients for suspected CSF leaks given the potentially dire sequelae.

## Conclusions

To our knowledge, this is the first case reported of a patient presenting with a spontaneous posterior-inferior clival wall CSF leak. With higher rates of meningitis associated with spontaneous CSF leaks in the clivus compared to other locations, the importance of early intervention and the push for clinicians to perform aggressive workups in suspected patients is prudent. A patient’s medical history of diseases associated with decreased bone density should also be taken into consideration as a potential factor that might contribute to spontaneous clival CSF leaks.
